# Validating Interactions of Pathogenic Proteins of *Staphylococcus aureus* and *E. coli* with Phytochemicals of *Ziziphus jujube* and *Acacia nilotica*

**DOI:** 10.3390/microorganisms11102450

**Published:** 2023-09-29

**Authors:** Wen Zou, Iram Hassan, Bushra Akram, Huma Sattar, Awais Altaf, Amjad Islam Aqib, Hassaan Bin Aslam, Mikhlid H. Almutairi, Kun Li

**Affiliations:** 1 MOE Joint International Research Laboratory of Animal Health and Food Safety, Institute of Traditional Chinese Veterinary Medicine, College of Veterinary Medicine, Nanjing Agricultural University, Nanjing 210095, China; 2 Center for Research in Molecular Medicine (CRiMM), Institute of Molecular Biology and Biotechnology (IMBB), The University of Lahore, Lahore 54590, Pakistanbushraakram1111@gmail.com (B.A.); huma_48biotech@yahoo.com (H.S.);; 3 Department of Medicine, Cholistan University of Veterinary and Animal Sciences, Bahawalpur 63100, Pakistan; amjadislamaqib@cuvas.edu.pk; 4 Institute of Microbiology, University of Veterinary and Animal Sciences, Lahore 54000, Pakistan; hassaan.aslam@uvas.edu.pk; 5 Zoology Department, College of Science, King Saud University, P.O. Box 2455, Riyadh 11451, Saudi Arabia; malmutari@ksu.edu.sa

**Keywords:** mastitis, *S. aureus*, *E. coli*, *Ziziphus jujube*, *Acacia nilotica*, extracts, GC–MS, molecular docking, antibacterial activity

## Abstract

This study focused on the assessment of the antimicrobial resistance of *Staphylococcus aureus* (*S. aureus*) and *Escherichia coli* (*E. coli*) isolated from bovine mastitis milk samples and the revealing anti-mastitis potential of phytocompounds of *Ziziphus jujube* and *Acacia nilotica* through molecular docking analysis. The mastitis milk samples were collected from various dairy farms for the isolation of the bacteria (*S. aureus* and *E. coli*) and their response to antibiotics. Ethanolic extracts of both plants were prepared. Their antibacterial activity was evaluated, and they were processed for phytochemical analysis after which, molecular docking analysis with pathogenic proteins of the bacteria was carried out. Parametric and non-parametric statistical analyses were performed to reach the conclusions of this study. The findings of the study revealed a higher drug resistance (≥40%) of *E. coli* against ampicillin, amikacin, and vancomycin, while *S. aureus* exhibited the highest resistance to ampicillin, erythromycin, and ciprofloxacin. The ethanolic extracts of the *Ziziphus jujube* and *Acacia nilotica* plants produced a ZOI between 18 and 23 mm against multidrug-resistant *S. aureus* and *E. coli*. Gas chromatography–mass spectrophotometry (GC–MS) was used to explore 15 phytocompounds from *Ziziphus jujube* and 18 phytocompounds from *Acacia nilotica*. The molecular docking analysis of 2cyclopenten−1-one,3,4,4 trimethyl and Bis (2ethylhexyl) phthalate of *Ziziphus jujube* showed a binding affinity of −4.8 kcal/mol and −5.3 kcal/mol and −5.9 kcal/mol and −7.1 kcal/mol against the DNA Gyrase and toxic shock syndrome toxin-1 proteins of *S. aureus* and *E. coli*, respectively. The suberic acid monomethyl ester of *Acacia nilotica* showed a binding affinity of −5.9 kcal/mol and −5 kcal/mol against the outer membrane protein A and Topoisomerase IV protein of *E. coli* and −5.1 kcal/mol and −5.8 kcal/mol against the toxic shock syndrome toxin-1 and Enterotoxin B proteins of *S. aureus*. Similarly, 2,2,4-trimethyl-1,3-pentanediol di-iso-butyrate showed a binding affinity of −6.5 kcal/mol and −5.3 kcal/mol against the outer membrane protein A and Topoisomerase IV of *E. coli* and −5.2 kcal/mol and −5.9 kcal/mol against the toxic shock syndrome toxin-1 and Enterotoxin B proteins of *S. aureus*, respectively. The study concluded that there was an increasing trend for the antimicrobial resistance of *S. aureus* and *E. coli*, while the *Ziziphus jujube* and *Acacia nilotica* plant extracts expressed significant affinity to tackle this resistance; hence, this calls for the development of novel evidence-based therapeutics.

## 1. Introduction

Antimicrobial resistance has become the leading cause of the treatment failure of infectious diseases all over the world. Resistance to antimicrobials continues to compromise disease-prevention strategies and to increase the production and economic losses in animal-based food systems. Among the common conditions manifested at the farm level, mastitis is one of the frequent conditions of dairy animals [1]. This disease affects the udders of the animals, leading to a drop in milk quantity and quality, which significantly affects the farm’s profitability [2]. Most infections last for an entire period of lactation or even the cow’s entire life span [3] and are connected to an annual economic loss of around USD 35 billion globally, while losses of USD 2 billion have been noted in the U.S. alone [4]. The data are scarce concerning Pakistan regarding the economic loss caused by mastitis. One of the studies from Pakistan, however, reported an annual loss of USD 3.75 million [5].

Mastitis is an inflammation of the mammary glands caused by physical, chemical, or infectious agents. The latter cause is further consisted of bacterial, viral, fungal, and protozoal agents, but bacterial etiology alone comprises more than 90% of mastitis cases. Among the bacterial etiology, *Staphylococcus aureus* (*S. aureus*) and *Escherichia coli* (*E. coli*) appear as the major causes of this condition all around the world [6,7]. An increase in the incidence of antimicrobial resistance among pathogens combined with the high costs of treatment have prompted the scientific community to look for substitutes with comparable efficacy to that of antibiotics [8]. In the quest to find alternatives to antibiotics, medicinal plant extracts could be a possible option to treat infections [9]. Unlike antibiotics, plant extracts are widely recognized as useful therapeutics [10] and have proven effective alternative to antibiotics against mastitis pathogens [11]. Mastitis has been traditionally treated with a range of plant species in Pakistan, which includes *Allium sativum*, *Bunium persicum*, *Oryza sativa*, and *Triticum aestivum*, but further alternatives are required to obtain satisfactory results [12]. 

*Ziziphus jujube* or Chinese dates and *Acacia nilotica* have been reported to have a broad-spectrum antibacterial activity against a variety of bacterial organisms [13]. The extracts of these plants have been used previously to alleviate or treat a variety of ailments, and the antimicrobial potential of their extracts could be extended to microbes that have developed resistance [9]. A major impediment in using plant-based extracts is that there is little knowledge about their chemical composition. The identification of phytochemicals in plant extracts is the core to understanding the antimicrobial activities of the various components. Moreover, the molecular docking of the active ingredients with the ligands of microbes could further reveal the antibacterial potential of plant-based extracts, which can further bridge the knowledge gap. The phytocompounds from the leaf extract of *Ziziphus jujube* are 2-Cyclopenten-1-one,3,4,4-trimethyl and Bis(2-ethylhexyl) phthalate, and those from *Acacia nilotica* include suberic acid monomethyl ester and 2,2,4-trimethyl-1,3-pentanediol di-iso-butyrate. These phytocompounds show greater negative binding energies, they follow Lipinski’s five rules, and have a strong repressive activity against mastitis. Proteins such as toxic shock syndrome toxin-1(2QIL), DNA Gyrase (5L3J), outer membrane protein A (IBXW), Enterotoxin B (3SEB), and Topoisomerase IV (3FV5) represent salient pathogenic characteristics of pathogens, which can serve as representative molecular docking profiles. Similarly, DNA Gyrase has been proven to be a valid target in the design of novel antibacterial drugs, while TSST-1 is a superantigen that stimulates the release of cytokines, causing the leakage of endothelial cells at low concentrations with a cytotoxic effect to the cells at high concentrations. Enterotoxin B helps stimulate cytokine release and inflammation. Outer membrane protein A enables intracellular survival, that is, the evasion from the body’s defenses. Topoisomerase IV helps create pores in the membranes of host cells (cell lysis) and catalyzes DNA double-strand breaks.

Hence, the current study was conducted with the aim to investigate the antibacterial activity of *Ziziphus jujube* and *Acacia nilotica* plant extracts against drug-resistant *S. aureus* and *E. coli* as well as the characterization of extracts via phytochemical analysis, using the GC–MS and the molecular docking techniques, to provide a prediction of the ligand–receptor complex structure.

## 2. Materials and Methods

### 2.1. Isolation and Antibiotic Susceptibility Profile of Mastitis Milk Samples

Milk samples (*n* = 200) of dairy cows suffering from either clinical or subclinical mastitis were aseptically collected as per standard guidelines [10], using convenient sampling techniques. For this purpose, various dairy farms (*n* = 20) in the district Lahore were included. From each dairy farm, *n* = 10 cows were selected based on their clinical presentation and complaints of reduced milk production and abnormal milk. The subclinical mastitis was decided based on somatic cell count greater than 200,000 or a positive California Mastitis Test (CMT). The animals that showed clinical signs were also included in this study. 

The milk samples were swabbed on a blood agar following incubation at 37 °C for 24 h. The typical colonies of *S. aureus* (golden yellow) and *E. coli* (grayish white and moist) were subcultured on mannitol salt agar and eosin methylene blue agar. Biochemical confirmation of the isolates was performed as per the guidelines of *Bergey’s’ Manual of Determinative Bacteriology* [7]. Further, the isolates were also confirmed via molecular analysis by targeting *nuc genes* (500 bp with primers as “forward primers 5′AAGGGCAATACGCAAAGAG 3′ and reverse primers 5′AAACATAAGCAACTTTAGCCAAG 3′”) in *S. aureus* (Figure S1) [14] and 23S rRNA gene (at 231 bp using primers E23S-F: ATCAACCGATTCCCCAGT; E23S-R: TCACTATCGGTCAGTCAGGAG) for *E. coli* (Figure S2) [15]. 

The isolates were processed further to assess their responses to a wider range of antibiotics (ampicillin, enrofloxacin, ciprofloxacin, trimethoprim, sulfamethoxazole, amikacin, oxytetracycline, vancomycin, erythromycin, linezolid, and azithromycin) as per Kirby Bauer’s method of disc diffusion. Bacterial cultures adjusted at 1–1.5 × 10^8^ colony forming units (CFU)/mL were swabbed on Mueller Hinton agar following which antibiotic discs were aseptically placed on agar. Incubation at 37 °C for 20–24 h was carried out, followed by a measurement of zones of inhibition (ZOI) using Vernier calipers and compared with standards.

### 2.2. Selection of Multiple Drug-Resistant Bacteria

The isolates of *S. aureus* and *E. coli* showing resistance to more than two classes of antibiotics were considered multiple drug-resistant (MDR) pathogens. To evaluate the antibacterial response of plant extracts, *n =* 05 MDR *S. aureus* and *E. coli* isolates were selected for their further use in the current study.

### 2.3. Preparation of Plant Extracts and Determination of Antimicrobial Activity

The leaves (2–3 kg) of *Ziziphus jujube* and flowers of *Acacia nilotica* were collected from Bahawalpur in 2021, an area where they are commonly found. The leaves were identified by a professional botanist and assigned the voucher number 3859 to aid in identification in the future. The leaves were shade-dried and pulverized into a fine powder. The powders (100 g) were emulsified in 250 mL of absolute ethanol for two weeks and filtered through Whatman paper (0.45 µm pore size). The solvent was evaporated through a rotary evaporator (PG-215-Rotary Water Bath-928) at 55 °C for 1 hour. The extracts were lyophilized in VaCo_2_ (ZIRBUS).

Ethanolic extracts were applied to MDR *S. aureus* and *E. coli* at various concentrations (125 mg/mL, 250 mg/mL, and 500 mg/mL of *Ziziphus jujube*, and 125 mg/mL and 500 mg/mL of *Acacia nilotica*). For this purpose, broth cultures of bacteria were adjusted to a concentration of 1-1.5 × 10^8^ CFU/mL. Filter paper discs were impregnated with various concentrations of ethanolic extracts and applied on to Mueller Hinton agar. The fresh growth of *E. coli* and *S. aureus* was swabbed before the application of discs. The plates were incubated at 37 °C for 24 h to observe the ZOI. Amoxicillin and erythromycin antibiotics purchased from the market were applied as positive controls. These antibiotics were mostly used on farms with greater efficacy, hence the reason for their selection in this study.

### 2.4. Phytochemical Analysis of Extracts

The gas chromatography mass spectrophotometry (GC–MS) technique was used for phytochemical analysis to identify the various compounds present in the ethanolic extracts. For this purpose, the GC–MS instrument (Agilent Technologies 7890A GC System, 5301 Stevens Creek Blvd, Santa Clara, CA, USA) fitted with an Elite-5ms fused silica column (30 m × 0.25 mm, film thickness 0.5 m) was operated, and Helium was used as a carrier gas. The leaf extract was mixed with ethanol (1:10 *v*/*v*). The injector volume was 1 L, and the injector temperature was set at 250 °C. The oven temperature was kept at 70 °C for 4 min before being raised to 310 °C at a rate of 5 °C/min for 10 min. All samples were evaluated in both full scan and selective ion scan modes (mass range of 40–510 atomic mass units). The temperatures of the injector inlet and transfer lines were 280 °C and 290 °C, respectively. Phytocompounds were obtained along with retention time and peak area percentage. A chromatogram was also achieved that showed the most numerous phytocompounds in the leaf extracts.

### 2.5. Derivation of Bacterial Pathogenic Protein

The three-dimensional structure of target proteins and their identification numbers were derived from the protein data bank (PDB). The proteins were prepared in the tool BIOVIA/Discovery studio 2021. For in silico protein purification, water and other molecules were removed from the structure. The proteins involved in mastitis were targeted as Topoisomerase IV (PDB ID: 3FV5), outer membrane protein A (PDB ID: 1BXW), and DNA Gyrase (PDB ID: 5L3J) for *E. coli*, and Enterotoxin B (PDB ID: 3SEB) and toxic shock syndrome toxin-1 (PDB ID: 2QIL) for *S. aureus* [16].

### 2.6. Selection of Effective Phytocompounds:

For the identification of the best ligand interaction, minimum binding energy needed to bind with a particular target protein was considered. From PubChem, two-dimensional structures of the active compounds present in extracts were downloaded and then changed into a Pbdqt file using Discovery Studio Vision. The ADMET rule (absorption, distribution, metabolism, excretion, and toxicity) was applied for the designing process. For this purpose, the compound was written in PubChem, and a canonical smile was taken and pasted in SwissADME to obtain ADMET properties. From SwissADME, phytocompounds were screened based on cytochrome inhibitors, whereas CYP inhibitors were favorable and performed activity in favor of drug.

### 2.7. Molecular Docking of Bacterial Proteins with Ligands of Extract

The aim of molecular docking was to predict the binding energy of small molecular ligand interactions with the binding site of the target protein. Compounds of 2-Cyclopenten-1-one,3,4,4-trimethyl-and-Bis(2-ethylhexyl) phthalate were docked with bacterial proteins (PDB ID: 5L3J for *E. coli* and 2QIL for *S. aureus*) that had high negative binding affinity. The molecular docking of phytocompounds was achieved with the help of PyRx software Version 0.9. The phytocompounds were docked by targeting the binding sites of proteins with grid box dimensions that were originated by fixing x, y, and z. In this way, the ligands were set on their binding energy, and bonding interaction strength was yielded between ligands and proteins. Similarly, suberic acid monomethyl ester and 2,2,4-trimethyl-1,3-pentanediol di-iso-butyrate from the extracts were docked with the 1BXW and 3FV5 proteins of *E. coli* and the 2QIL and 3SEB proteins of *S. aureus*. Blind docking was applied to these compounds as they show greater negative binding energies [17].

The docked complex was applied to find the ligand–protein interactions. Discovery Studio was used to visualize the protein–ligand complex obtained by docking through PyRx. In the Discovery Studio, binding energies, receptor–ligand interaction, H-bond, and the distance-like properties were visualized, which helped to screen phytocompounds that have the highest antibacterial activity and could be a candidate for drug design.

## 3. Results

### 3.1. Antimicrobial Resistance Profile of S. aureus and E. coli

The responses of *E. coli* and *S. aureus* against antibiotics showed considerable resistance (Table 1). The study found that more than 40% of *E. coli* isolates were resistant to ampicillin, trimethoprim, sulfamethoxazole, amikacin, and vancomycin. Around 40% of the *S. aureus* isolates were found resistant to ampicillin, ciprofloxacin, and erythromycin. The study showed a higher percentage of intermediate-susceptible isolates of *S. aureus* as well as *E. coli*. It was noteworthy that *E. coli* isolates were resistant to the majority of the antibiotics (equal to or more than 30%), except for erythromycin, oxytetracycline, and ciprofloxacin. In the case of *S. aureus*, amikacin and linezolid were found to be the most effective candidates. The percentage-sensitive isolates remained more than 40% against all antibiotics except ampicillin (Table 1).

### 3.2. Response of Multiple Drug-Resistant E. coli and S. aureus against Ethanolic Plants’ Extracts 

**Figure 1:**
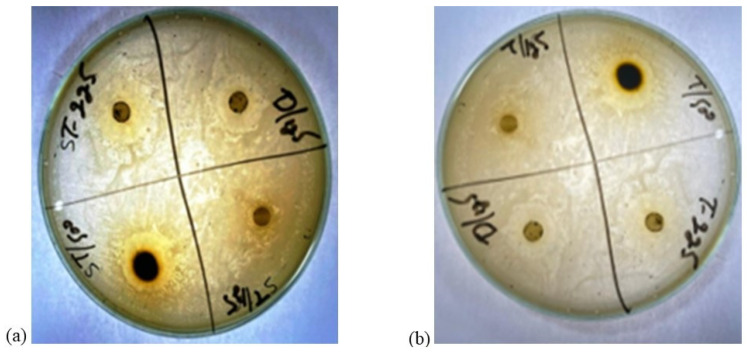
Zones of inhibition of leaf extract of *Ziziphus jujube* plant. (**a**) zone of inhibition(mm) of *Ziziphus jujube* for *S. aureus* (**b**) zone of inhibition (mm) of *Ziziphus jujube* for *E. coli*.

**Figure 2:**
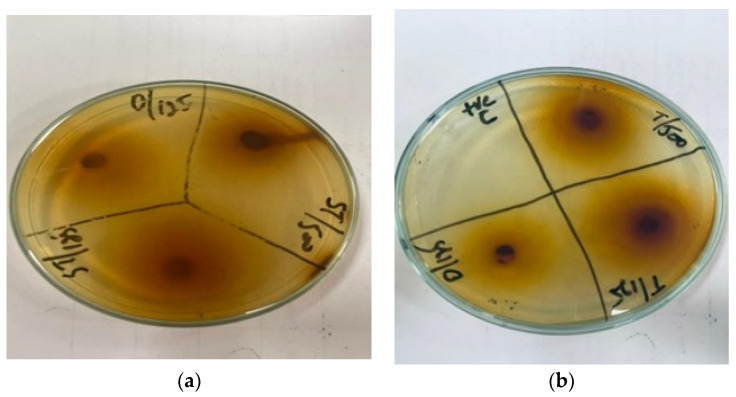
Antibacterial activity of *Acacia nilotica* flower’s extract at 500 and 125 mg/mL of plant’s concentration and Amoxicillin concentration 125 mg. (**a**) Zone of inhibition (mm) of *Acacia nilotica* for *S. aureus;* (**b**) zone of inhibition (mm) of *Acacia nilotica* for *E. coli*.

The ZOI shown by the leaf extract of *Ziziphus jujube* revealed that plants pose antibacterial properties against Gram-positive (*S. aureus*) and Gram-negative (*E. coli*) bacteria (Table 2, Figure 1). The ethanolic leaf extract of *Ziziphus jujube* showed ZOI values of 21 mm, 19 mm, and 18 mm, and 23 mm, 21 mm, and 18 mm at concentrations of 500 mg, 225 mg, and 125 mg for *S. aureus* and *E. coli*, respectively. Ethanolic extracts of *A. nilotica* showed 20 mm and 18 mm ZOI at concentrations of 500 mg and 125 mg, respectively, against *S. aureus*, while amoxicillin showed a 17 mm ZOI at 125 mg concentration. Against *E. coli*, the same extract concentrations of 500 mg and 125 mg were used, which showed ZOI values of 23 mm and 21 mm, respectively (Table 2, Figure 2). 

### 3.3. Phytochemical Analysis of Plant Extract

**Figure 3:**
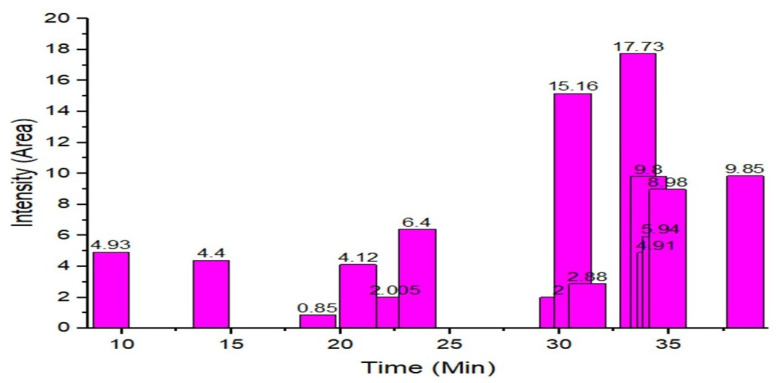
GC–MS chromatogram of ethanolic phytocompounds of *Ziziphus jujube* leaf extract.

**Figure 4:**
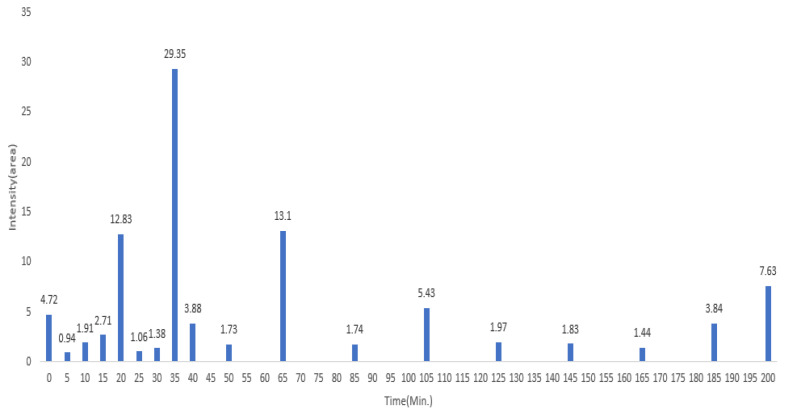
GC–MS chromatogram of ethanolic phytocompounds of *Acacia nilotica* flowers extract.

The GC–MS results of *Ziziphus jujube* revealed that phytol was the most abundant compound (with a 17.73% intensity) detected at 35 min (Table 3) on the GC–MS column, and n-hexadecanoic acid with a 15.16% intensity was detected at 30 minutes (with the attribute of being antibacterial). Fifteen peaks were detected in GC–MS chromatogram for ethanolic extracts of the leaf extract of *Ziziphus jujube* (Figure 3). Eighteen peaks were detected in GC–MS chromatogram for ethanolic extracts of the flowers of *Acacia nilotica* (Figure 4, Table 4). 

2-Cyclopenten-1-one,3,4,4-trimethyl-, and Bis(2-ethylhexyl)phthalate were two screened compounds fulfilling ADMET properties (Table 5). Two compounds (2,2,4-trimethyl-1,3-pentanediol diisobutyrate and suberic acid monomethyl ester) showed the highest antibacterial activity and followed Lipinski’s five rules (Table 6). The former molecule has a molecular weight of 124.18 g/mol and has high GI absorption along with antibacterial activity. The latter molecule was attributed to having a molecular weight of 376.53 g/mol and a high GI absorption with antibacterial activity. The Lipinski’s rule of five was employed for all 13 ligands, which was different for each ligand. Out of the 13 phytocompounds, 2 compound (2-Cyclopenten-1-one,3,4,4-trimethyl- and Bis(2-ethylhexyl)) phthalates were screened out for molecular docking (Table 7).

Same is the case with phytochemical analysis of *Acacia nilotica* based on ADMET parameters as indicated in Table 8.

### 3.4. Derivation of Bacterial Proteins

The study derived DNA Gyrase (5L3J) protein of *E. coli* because of its proven action to serve as target for drug designs (Table 9). Similarly, toxic shock syndrome toxin-1(TSST-1 2QIL) of *S. aureus* proteins were derived with the fact that it being superantigen could provoke cytokines storm and cytotoxic effects to cells when used. Similarly, 1BXW outer membrane protein A and topoisomerase IV from *E. coli;* and Toxic shock syndrome toxin-1, and 3SEB Enterotoxin B from *S. aureus* protein were found (Table 10).

### 3.5. Molecular Docking of Bacterial Protein with Ligands of Phytocompounds

All the compounds showed different binding energies when docked with the ligands of phytocompounds. The binding energies of phytocompounds of *Ziziphus jujube* leaf extracts with *S. aureus* and *E. coli* proteins showed higher minus-binding energies, indicating a greater stability of the compounds (Table 11). 2-Cyclopenten-1-one,3,4,4-trimethyl showed −4.8 kcal/mol and −5.3 kcal/mol binding energies with proteins from *E. coli* and *S. aureus*, respectively, while Bis(2-ethylhexyl) phthalate showed −5.9 kcal/mol and −7.1 kcal/mol binding energies against *E. coli* and *S. aureus* proteins, respectively (Table 11). These energies indicate that the screened phytocompounds have sufficient suppressive ability against mastitis and hold a greater stability for their sustained efficacy.

**Figure 5:**
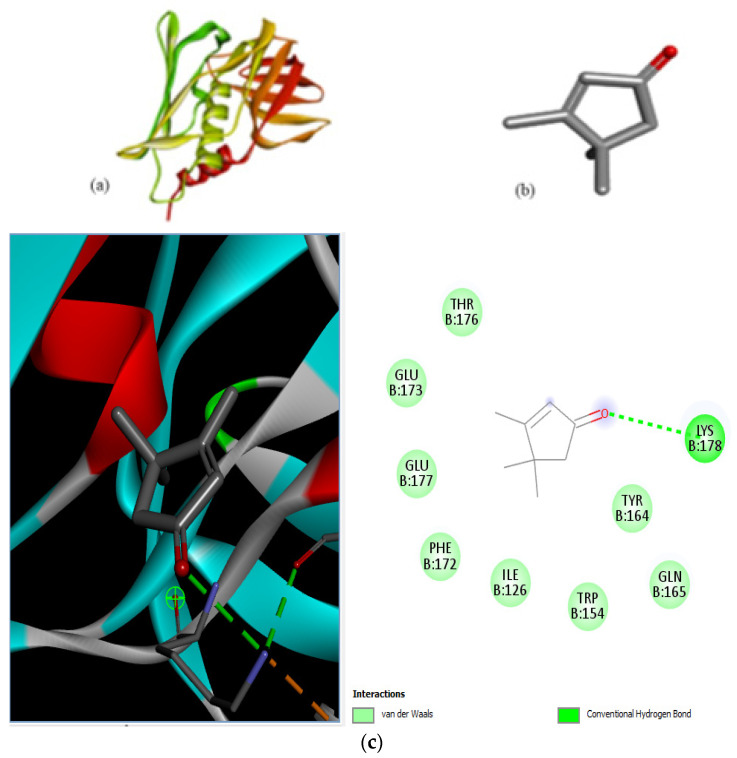
Molecular docking of *Staphylococcus aureus* protein with cyclopentene ligands of *Ziziphus jujube* plant. (**a**) Toxic shock syndrome toxin-1 target protein PDB structure. (**b**) 2-Cyclopenten-1-one,3,4,4-trimethyl-ligand PDB structure. (**c**) 2-Cyclopenten-1-one,3,4,4-trimethyl showing interaction with target protein and 2D structure showing interaction with residues.

**Figure 6:**
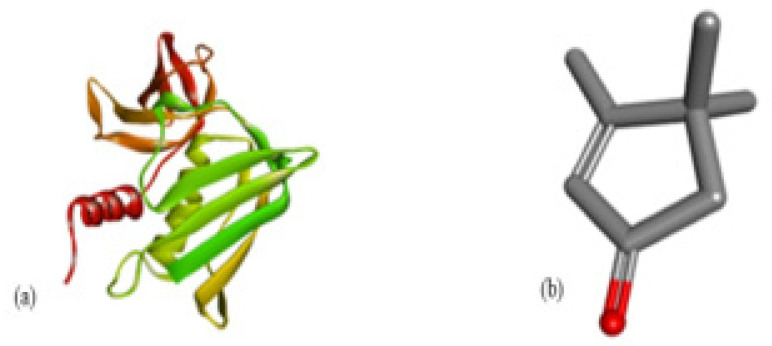

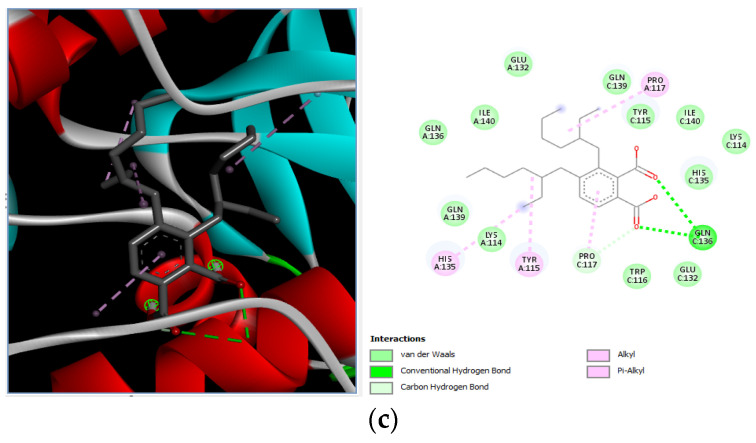
Molecular docking of *Staphylococcus aureus* protein with phthalate ligands of *Ziziphus jujube* plant. (**a**) Toxic shock syndrome toxin-1 target protein PDB structure. (**b**) Bis(2-ethylhexyl) phthalate ligand PDB structure. **(c)** Bis(2-ethylhexyl) phthalate—showing interaction with target protein and 2D structure showing interaction with residues.

**Figure 7:**
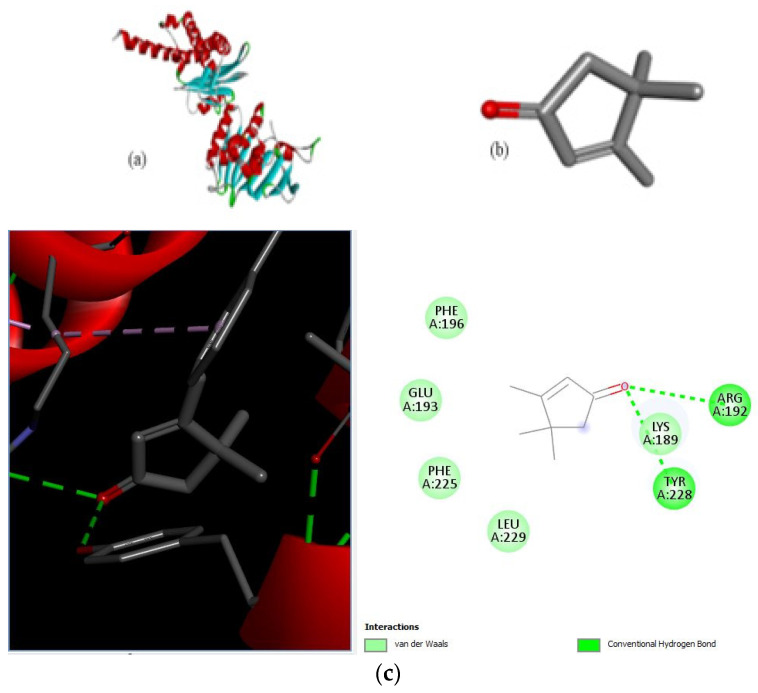
Molecular docking of *E. coli* protein with phthalate ligands of *Ziziphus jujube* plant. (**a**) DNA Gyrase target protein PDB structure. (**b**) 2-Cyclopenten-1-one,3,4,4-trimethyl-ligand PDB structure. (**c**) 2-Cyclopenten-1-one,3,4,4-trimethyl showing interaction with target protein and 2D structure showing interaction with residues.

**Figure 8:**
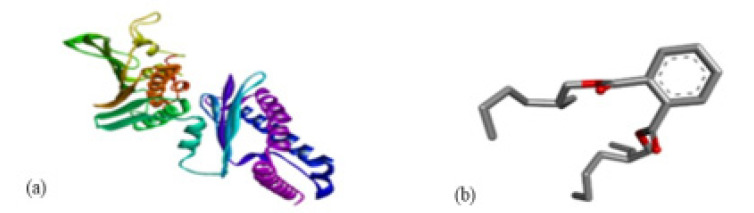

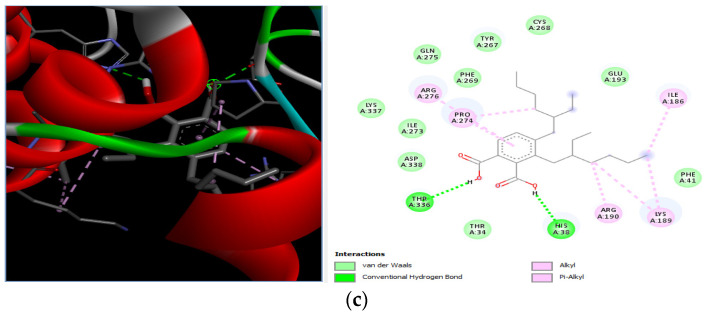
Molecular docking of *E. coli* protein with phthalate ligands of *Ziziphus jujube* plant. (**a**) DNA Gyrase target protein PDB structure. (**b**) Bis(2-ethylhexyl) phthalate ligand PDB structure. (**c**) Bis(2-ethylhexyl) phthalate—showing interaction with target protein and 2D structure showing interaction with residues.

The docking interaction of toxic shock syndrome toxin (TSST-1) with the 2-Cyclopenten-1-one,3,4,4-trimethyl-ligand PDB structure (Figure 5) and Bis(2-ethylhexyl) phthalate ligand PDB structure of TSST-1 protein (Figure 6) revealed strong interactions with each other and with residue molecules. Similarly, the docking interaction of DNA Gyrase target protein PDB structure with 2-Cyclopenten-1-one,3,4,4-trimethyl-ligand PDB structure (Figure 7) and Bis(2-ethylhexyl) phthalate ligand PDB structure (Figure 8) showed unique interactions with each other and other residue molecules.

**Figure 9:**
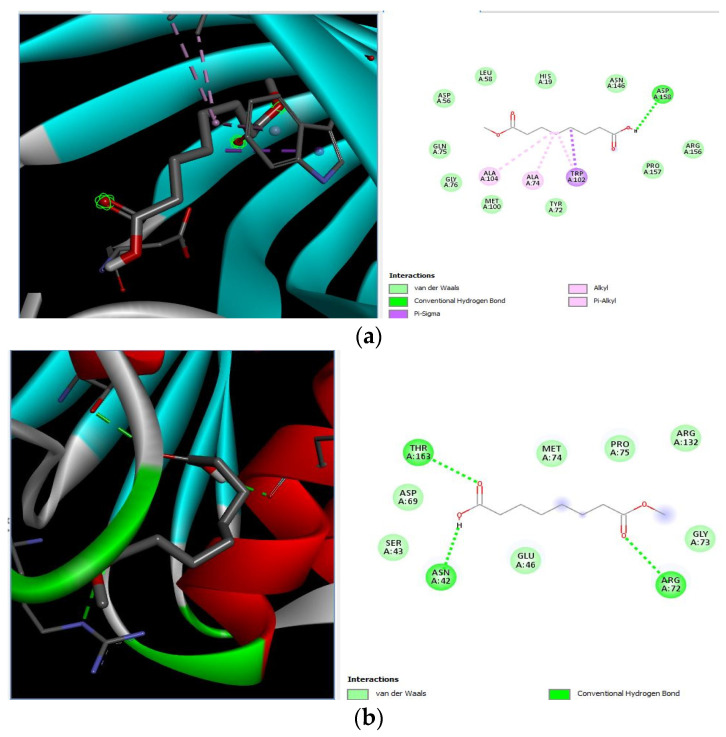

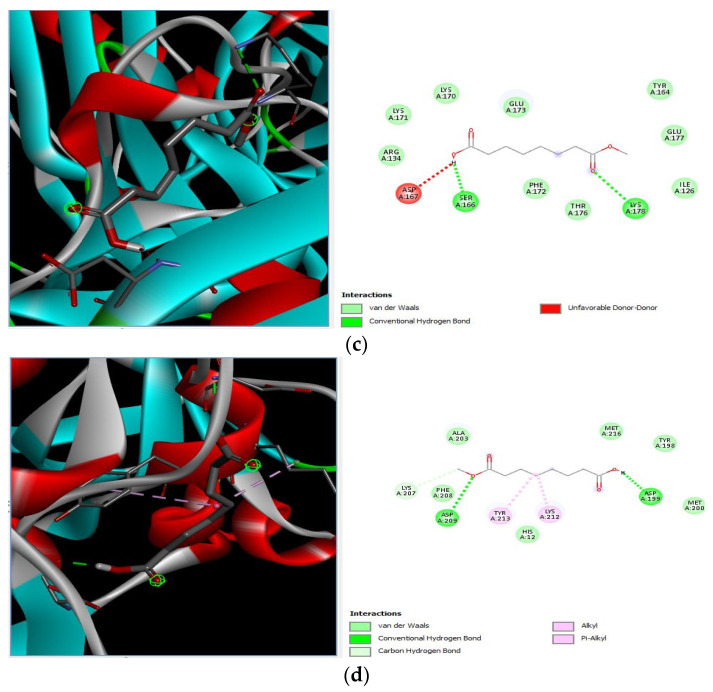
Molecular docking of suberic acid monomethyl ester with *S. aureus* and *E. coli* proteins: (**a**) 2D and 3D representations of greater negative binding energy between suberic acid monomethyl ester and outer membrane protein A (PDB ID: IBXW). (**b**) Docking interaction between suberic acid monomethyl ester and Topoisomerase IV (PDB ID: 3FV5). (**c**) Docking interaction between suberic acid monomethyl ester and toxic shock syndrome toxin-1 (PDB ID: 2QIL). (**d**) 2D and 3D representations of greater negative binding energy between suberic acid monomethyl ester and Enterotoxin B (3SEB).

**Figure 10:**
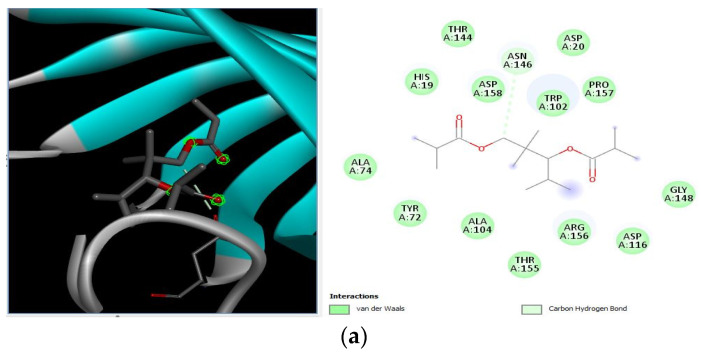

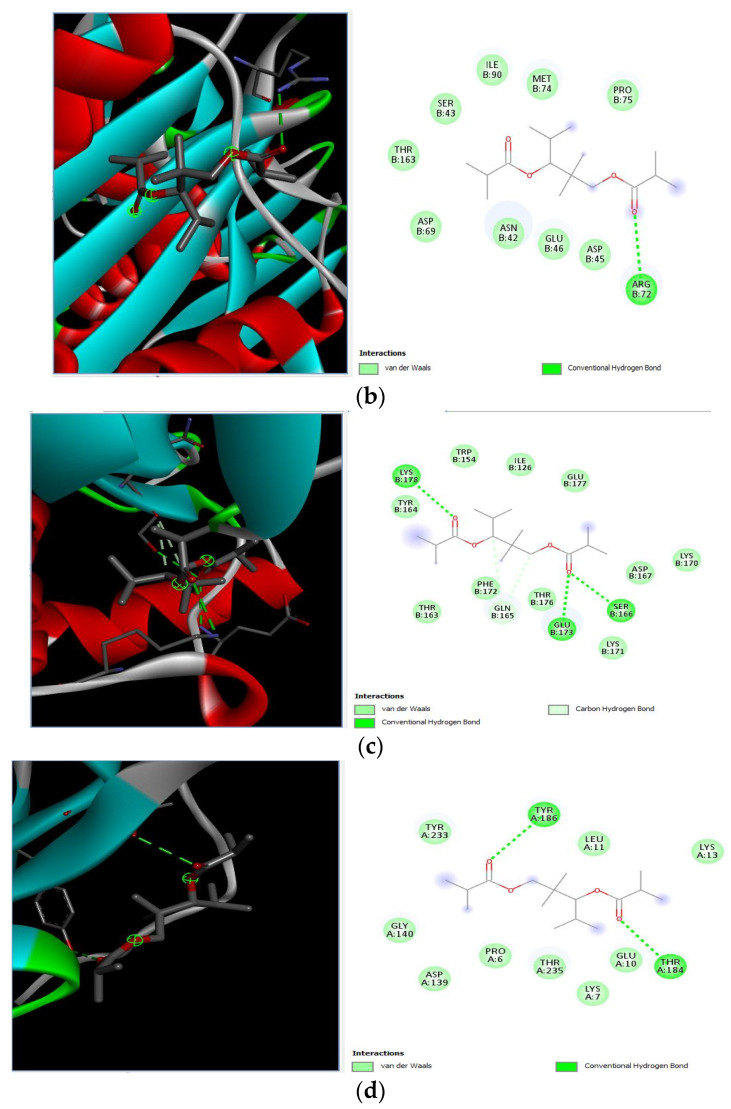
Molecular docking of 2,2,4-trimethyl-1,3-pentanediol di-isobutyrate with *S. aureus* and *E. coli* proteins. (**a**) 2D and 3D representations of greater binding energy between 2,2,4-trimethyl-1,3-pentanediol di-isobutyrate and outer membrane protein A. (**b**) Docking interaction between 2,2,4-trimethyl-1,3-pentanediol di-isobutyrate and Topoisomerase IV. (**c**) Docking interaction between 2,2,4-trimethyl-1,3-pentanediol di-isobutyrate and toxic shock syndrome toxin-1. (**d**) 2D and 3D representations of greater binding energy between 2,2,4-trimethyl-1,3-pentanediol di-isobutyrate and Enterotoxin B.

Different binding energies when the proteins of *E. coli* and *S. aureus* were docked with the binding sites of ligands are shown in Figure 9 and Figure 10. The binding energies of suberic acid monomethyl ester were higher when docked against outer membrane protein A (−5.9 kcal/mol) of *E. coli* and Enterotoxin B (−5.8 kcal/mol) of *S. aureus* and when following Lipinski’s five rules, while Topoisomerase IV and TSST-1 showed lower binding energies at −5 kcal/mol and −5.1 kcal/mol, respectively. 2,2,4-trimethyl-1,3-pentanediol di-isobutyrate showed higher negative binding energies (−6.5 kcal/mol) for *E. coli* protein (PDB ID: IBXW) and (−5.9 kcal/mol) against Enterotoxin B, while lower binding energies were evident for Topoisomerase IV and TSST-1 (−5.3 kcal/mol and −5.2 kcal/mol, respectively). These two phytocompounds showed higher negative binding energies and followed Lipinski’s five rules, and had strong repressive action against mastitis, while other compounds possessed less than −5 kcal/mol binding energies and were found to be less stable (Table 11).

## 4. Discussion

The studies have proven that the regular use of antibiotics results in the development of antimicrobial resistance (AMR) among core mastitis pathogens like *E. coli* and *S. aureus*. Moreover, the use of plant extracts has been found to be effective in achieving optimum control over AMR mastitis pathogens. One of the studies conducted in France has concluded antimicrobial resistance against three primary isolated bacteria (*Streptococcus uberis*, *E. coli*, and Coagulase-positive *staphylococci*) isolated from dairy cattle and found high levels of resistance against commonly used antimicrobials [26]. These findings are in accordance with our results, where a higher resistance against antibiotics was found. The bacterium *S. aureus* is one of the reasons for chronic mastitis that could be prevented by on-time diagnosis, ensuring good farm management, and improving udder hygiene. The production of metabolizing enzymes and toxins by *S. aureus* that damage the milking tissue resulted in a deeper penetration of bacteria into the tissue. Hence, it became difficult for antibiotics to reach the affected tissue at the minimum inhibitory concentration (MIC), leading to the development of AMR [13]. Therefore, alternatives to antibiotics that could suppress bacterial capability to colonize are recommended. Moreover, the overuse of antibiotics to manage AMR contributes to the development of multidrug resistance, which has severe animal and public health implications.

In a previous study, *Ziziphus jujube* showed effective antibacterial activity against Gram-positive and Gram-negative bacteria such as *S. aureus* and *E. coli* [6], which is in line with the current study. Similar results were reported by another study that concluded the antibacterial activity of *Ziziphus* leaves extract at 10mg/mL against *S. aureus, Listeria. monocytogenes*, *Salmonella typhimurium*, and *E. coli* with ZOI ranging from 10 mm to 14.2 mm for various plant extracts. Likewise, another study reported a zone of inhibition of *Ziziphus* leaf extract, ranging between 7 mm and 15 mm against *S. aureus*, *E. coli*, and *S. typhimurium*. These antibacterial properties of the plant extracts are due to the presence of enriched constituents like polyphenols, flavonoids, and tannins [27]. The higher contents of phenols in the plant extracts were detected in the current study. These findings are consistent with a previously reported study, where high concentrations of phenolic contents in the leaves of *Ziziphus jujube* plants were found with potential antibacterial activity [28]. Similarly, higher concentrations of total flavonoids were detected as found by Song *et al*. [29]. A higher concentration of phenols in the leaves might be due to the presence of phenolic compounds in vacuoles of the colored tissues of plants [30]. The presence of phenolic compounds with antibacterial activity as identified in our study has also been determined in coffee and other plant species. The isolated compounds from the phenolic class were quinic acid, gallic acid, catechin, chlorogenic acid, 4-0-caffeoylquinic acid, caffeic acid, syringic acid, rutin, 1,3-di-O-caffeoyulquinic acid, epicatechin, *p*-coumaric acid, trans-ferulic acid, hyperoside, and others [31]. Phenolic compounds like 3,4-dihydroxyphenyl ethanol glucoside have been proven to express the highest bacteriostatic activity against *Bacillus cereus*, *S. typhimurium*, *S. aureus*, and *E. coli*.

Our results indicated that *Ziziphus jujube* has effective antibacterial activity against Gram-positive and Gram-negative bacteria such as *S. aureus* and *E. coli*, as was also found by Kher *et al*. [32]. Around 15 phytocompounds from *Ziziphus jujube* leaf extract and 18 phytocompounds from the flower extract of *Acacia nilotica* were obtained from GC–MS and screened by Swiss ADME on the basis of CYP inhibitors. The following phytocompounds were selected for molecular docking that have an area sum% 2-cyclopenten-1-one,3,4,4-trimethyl (4.93%), Bis(2-ethylhexyl) phthalate (5.94%), suberic acid monomethyl ester (2.71%), and 2,2,4-trimethyl-1-3-pentanediol di-isobutyrate (1.06%). The phytocompounds selected have CYP inhibitors favorable for proteins and perform activity in favor of drugs, while the approach to select drugs on the basis of CYP inhibitors were in agreement with other studies [16]. The compound 3,4-dihydroxyphenyl ethanol glucoside expressed the highest bacteriostatic activity against *Bacillus cereus*, *Salmonella typhimurium*, *S. aureus*, and *E. coli* [16]. Among other compounds, flavonoids are believed to be the most active compounds that act by intercalation into DNA, leading to frameshift changes [33], whereas another study [34] has reported the promotion of Topoisomerase IV-dependent DNA cleavage. Moreover, geographical area could be an important factor causing variation in the phenolic composition of these plants’ extracts, but this factor could not create any bias in our study as all plants were sourced from a region with a uniform climate. Given the strong antibacterial potential of extracts of *Acacia nilotica* flowers and *Ziziphus jujube* leaves, it could be stated that the extracts of these plants could be used as an alternative to antibiotics if used in optimum concentrations. The use of *Acacia nilotica* and *Ziziphus jujube* extracts to prevent, control, or treat many infectious diseases of bacterial origin has been well recognized in homeopathic medicine [7]. *Acacia nilotica* flowers and *Ziziphus jujube* leaf extracts have shown greater antibacterial activity comparable to commonly used antibacterials on dairy farms, providing an auspicious tool for mastitis control.

Extracts and phytocompounds from *Humulus lupulus* showed significant activity against *S. aureus* compared to their response against *Lactobacillus acidophilus* [35]. Similarly, the antimicrobial activity of the tannins of the chestnut extract was noted, while this response was pronounced in the case of *E. coli*, and hence reflected the availability of potential alternative to antimicrobials [36]. The In silico inverse molecular docking procedures have been used previously to measure antimicrobial activity of rosemary extracts [37]. Xanthohumol and its metabolites were effectively docked with most human pathogenic proteins to aid in anticancer and antimicrobial activities [38]. In extension to the previous study [37], protein–ligand complexes were observed for K-RAS, HIV-1, and factor X was found to explore the simulation of molecular dynamics and free energy calculations. Carnosic acid and rosmanol were found to be significant binders with the HIV-1 protease, while carnasol showed prominent binding with the protein K-RAS [39].

## 5. Conclusions

The study found an increasing resistance in *S. aureus* and *E. coli* against a wider range of antibiotics from various classes depicting the narrowing window of the efficacy of antibiotics available at present. The plant extracts of *Ziziphus jujube* and *Acacia nilotica* have shown antibacterial activity against multiple drug-resistant *S. aureus* and *E. coli* equivalent to the positive control (erythromycin and amoxicillin). The extract of *Ziziphus jujube* and *Acacia nilotica* plants have a variety of phytocompounds that agreed with ADMET and Lipinski rules and have proven their antibacterial activity. The molecular docking of the two most appropriately screened phytocompounds 2-cyclopenten-1-one,3,4,4-trimethyl- and Bis(2-ethylhexyl) phthalate showed a greater binding affinity with pathogenic proteins of *S. aureus* (toxic shock syndrome toxin-1) and *E. coli* (DNA Gyrase). Similarly, suberic acid monomethyl ester and 2,2,4-trimethyl-1,3-pentanediol di-iso-butyrate of *Acacia nilotica* proved to be effective molecular ligands with pathogenic proteins. The study thus reached to the conclusion that there was an increase in resistance against antibiotics, while a greater antibacterial scope was noted in using phytocompounds of plant extracts that can pave the way to develop alternative therapeutics from natural resources.

## Figures and Tables

**Table 1:** Response of *S. aureus* and *E. coli* against antibiotics.

Antibiotics Name	*E. coli*			*S. aureus*		
	R (%)	I (%)	S (%)	R (%)	I (%)	S (%)
Ampicillin	50	20	30	40	30	30
Enrofloxacin	30	10	60	30	20	50
Ciprofloxacin	20	10	70	40	10	50
Trimethoprim Sulfamethoxazole	40	20	40	30	20	50
Amikacin	50	20	30	20	10	70
Oxytetracycline	20	30	50	30	20	50
Vancomycin	40	20	40	30	10	60
Erythromycin	10	20	70	40	20	40
Linezolid	20	30	50	20	20	60
Azithromycin	30	30	40	30	20	50

R = Resistant; I = Intermediate; S = Sensitive.

**Table 2:** Zone of inhibition (mm) for *E. coli* and *S. aureus* against extracts of plants.

Effect of *Ziziphus jujube*			
Bacteria	Extract Concentration (mg/mL)	Inhibition Zone Diameter (mm)	Erythromycin * (125 mg/mL)
*S. aureus*	500 mg/mL 225 mg/mL 125 mg/mL	21 mm 19 mm 18 mm	17 mg/mL
*E. coli*	500 mg/mL 225 mg/mL 125 mg/mL	23 mm 21 mm 18 mm	15 mg/mL
**Efficacy of *Acacia nilotica***			
Isolates	Extract Concentration (mg/mL)	Inhibition Zone Diameter (mm)	Amoxicillin (125 mg/mL)
*S. aureus*	500 mg/mL 125 mg/mL	20 mm 18 mm	17 mg/mL
*E. coli*	500 mg/mL 125 mg/mL	23 mm 21 mm	15 mg/mL

* The antibiotic disc was for clinical purpose and was purchased from a medical outlet.

**Table 3:** GC–MS results showing phytocompounds in ethanolic extract of *Ziziphus jujube*.

Sr. No.	Peak Compounds	2D Structure	Molecular Weight (g/mol)	Retention Time (min)	Area Sum (%)	Formula	Classification
1	2-Cyclopenten-1-one,3,4,4-trimethyl-	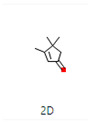	124.18 g/mol	9.524 m	4.93%	C_8_H_12_O	Reported to exhibit the higher tumor-specific cytotoxicity against oral human normal and tumor cells
2	Benzenepropanal, 4-(1,1-dimethylethyl)-	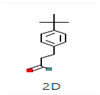	190.28 g/mol	14.107 m	4.40%	C_13_H_18_O	Not reported
3	Oxalic acid, dodecyl isobutyl ester	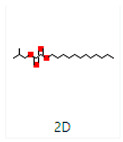	300.43 g/mol	18.954 m	0.85%	C_17_H_32_O_4_	Not reported
4	2,4-Di-tert-butylphenol	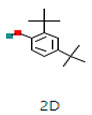	192.30 g/mol	20.808 m	4.12%	C_13_H_20_O	It has a role as a bacterial metabolite, an antioxidant, and a marine metabolite. It is an alkylbenzene and a member of phenols
5	Azelaic acid	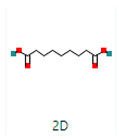	172.22 g/mol	22.490 m	2.05%	C_9_H_16_O_3_	It has a role as an antibacterial agent, an antineoplastic agent, a dermatologic drug, and a plant metabolite
6	Pentadecanoic acid, 14-methyl-, methyl ester	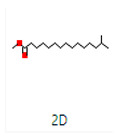	256.42 g/mol	29.946 m	2.00%	C_16_H_32_O_2_	Not reported
7	n-Hexadecanoic acid	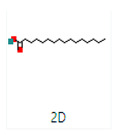	240.42 g/mol	30.621 m	15.16%	C_16_H_32_O	It has a role as an EC 1.1.1.189 (prostaglandin-E2 9 reductase) inhibitor, a plant metabolite, a Daphnia magna metabolite, and an algal metabolite
8	Octadecanoic acid, 9,10-dihydroxy, methyl ester	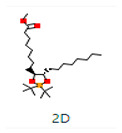	456.77 g/mol	31.285 m	2.88%	C_26_H_52_O_4_Si	Not reported
9	Phytol	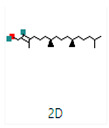	282.50 g/mol	33.591 m	17.73%	C_19_H_38_O	It has a role as a plant metabolite, a schistosomicide drug, and an algal metabolite. It is a diterpenoid and a long-chain primary fatty alcohol
10	9,12,15-Octadecatrien-1-ol, (Z,Z,Z)-	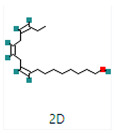	248.45 g/mol	34.054 m	9.80%	C_18_H_32_	It has a role as an antibacterial agent
11	Octadecanoic acid	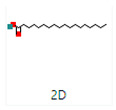	268.48 g/mol	34.409 m	4.91%	C_18_H_36_O	It has a role as a plant metabolite, a human metabolite, a Daphnia magna metabolite, and an algal metabolite
12	Bis(2-ethylhexyl) phthalate	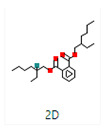	376.53 g/mol	34.621 m	5.94%	C_23_H_36_O_4_	Di(2-ethylhexyl) phthlate (DEHP) is a manufactured chemical that is commonly added to plastics to make them flexible
13	Methyl 9-cis,11-trans-octadecadienoate	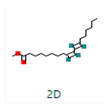	280.45 g/mol	34.947 m	8.98%	C_18_H_32_O_2_	Not reported

**Table 4:** GC–MS results showing phytocompounds in ethanol extract of *Acacia nilotica*.

Sr. No.	Peak Compounds	2D Structure	Formula	Molecular Weight (g/mol)	Retention Time (min)	Area Sum (%)	Classification
1	Nonanoic acid	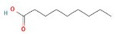	C_9_H_18_O_2_	158.24 g/mol	12.528 m	4.72%	Antibacterial (reduce bacterial translocation)
2	Methyl 8-oxooctanoate	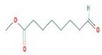	C_9_H_16_O_3_	172.22 g/mol	14.645 m	0.94%	Anticancer
3	Nonanoic acid, 9-oxo-, methyl ester	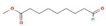	C_10_H_18_O_3_	186.25 g/mol	18.159 m	1.91%	Antimicrobial, antioxidant (cancer)
4	Suberic acid monomethyl ester	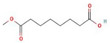	C_9_H_16_O_4_	188.22 g/mol	19.875 m	2.71%	Antibacterial (growth inhibition)
5	Nonanedioic acid, dimethyl ester	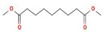	C_11_H_20_O_4_	216.27 g/mol	22.530 m	12.83%	Bacteriostatic, antioxidant (inhibitor of mitochondrial oxidoreductases)
6	2,2,4-Trimethyl-1,3-pentanediol diisobutyrate	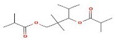	C_16_H_30_O_4_	286.41 g/mol	22.730 m	1.06%	Antibacterial
7	1,2-Benzenedicarboxylic acid, bis(2-methylpropyl) ester	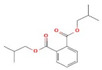	C_16_H_22_O_4_	278.34 g/mol	28.881 m	1.38%	Antibacterial, Antimicrobial
8	Hexadecanoic acid, methyl ester	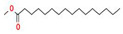	C_17_H_34_O_2_	270.5 g/mol	29.951 m	29.35%	Antiandrogenic (autolysis of membrane structure, especially mitochondria)
9	n-Hexadecanoic acid	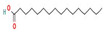	C_16_H_32_O_2_	256.42 g/mol	30.638 m	3.88%	Antibacterial (hydrolysis of membrane phospholipid)
10	Cyclic octaatomic sulfur	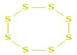	S_8_	256.5 g/mol	32.629 m	1.73%	Bacterial metabolite
11	11-Octadecenoic acid, methyl ester	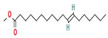	C_19_H_36_O_2_	296.5 g/mol	33.362 m	13.10%	Antioxidant
12	9-Octadecenoic acid (z)-, methyl ester	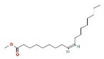	C_19_H_36_O_2_	296.5 g/mol	33.459 m	1.74%	Antimicrobial
13	Methyl stearate	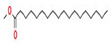	C_19_H_38_O_2_	298.5 g/mol	33.802 m	5.43%	Anticancer (unfolding of protein)
14	9-Octadecenoic acid, (E)-	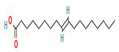	C_18_H_34_O_2_	282.5 g/mol	34.026 m	1.97%	Antiviral, antioxidant
15	Methyl-9-cis,11-trans-octadecadienoate	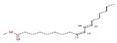	C_19_H_34_O_2_	294.5 g/mol	34.117 m	1.83%	Anticarcinogenic
16	Octadecanoic acid	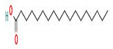	C_18_H_36_O_2_	284.5 g/mol	34.420 m	1.44%	Antibacterial
17	Octadecanoic acid, 10-oxo-, methyl ester	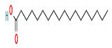	C_19_H_36_O_3_	312.5 g/mol	37.287 m	3.84%	Antibacterial, Antitumor
18	1H-Pyrrol-1-yloxy, 3(aminocarbonyl)-2,5-dihydro-2,2,5,5-tetramethyl	Has no 2D structure	C_9_H_15_N_2_O_2_	183.2276 g/mol	38.500 m	7.63%	

**Table 5:** Phytocompounds of *Ziziphus jujube* showing ADMET properties for molecular docking.

Sr. No.	Phytocompounds	Molecular Formula	Mode of Action	Lipinski Rule	ADMET Rule
1	2-Cyclopenten-1-one,3,4,4-trimethyl-	C_8_H_12_O	Antibacterial	Yes	Molecular weight = 124.18 g/mol GI absorption = High Water solubility = soluble Leadlikeness = No TPSA = 17.07 Å^2^ CYP1A2 inhibitor = No CYP2C19 inhibitor = No CYP2C9 inhibitor = No CYP2D6 inhibitor = No CYP3A4 inhibitor = No Consensus Log *P*_o/w_ = 1.80
2	Benzenepropanal, 4-(1,1-dimethylethyl)-	C_13_H_18_O	Skin allergic	Yes	Molecular weight = 190.28 g/mol GI absorption = High Water solubility = Moderately soluble Leadlikeness = No TPSA = 17.07 Å² CYP1A2 inhibitor = No CYP2C19 inhibitor = No CYP2C9 inhibitor = No CYP2D6 inhibitor = Yes CYP3A4 inhibitor = No Consensus Log *P*_o/w_ = 3.21
3	Oxalic acid, dodecyl isobutyl ester	C_17_H_32_O_4_		Yes	Molecular weight = 300.43 g/mol GI absorption = High Water solubility = Moderately soluble Leadlikeness = No TPSA = 52.60 Å² CYP1A2 inhibitor = Yes CYP2C19 inhibitor = No CYP2C9 inhibitor = No CYP2D6 inhibitor = No CYP3A4 inhibitor = No Consensus Log *P*_o/w_ = 4.83
4	2,4-Di-tert-butylphenol	C_13_H_20_O	Antibacterial; Anti-inflammatory; Antiviral	Yes	Molecular weight = 192.30 g/mol GI absorption = High Water solubility = Soluble Leadlikeness = No TPSA =20.23 Å^2^ CYP1A2 inhibitor = No CYP2C19 inhibitor = No CYP2C9 inhibitor = No CYP2D6 inhibitor = Yes CYP3A4 inhibitor = No Consensus Log *P*_o/w_ = 3.69
5	Azelaic acid	C_9_H_16_O_3_	Anti-inflammatory	Yes	Molecular weight = 172.22 g/mol GI absorption = High Water solubility = Soluble Leadlikeness = No TPSA = 54.37 Å^2^ CYP1A2 inhibitor = No CYP2C19 inhibitor = No CYP2C9 inhibitor = No CYP2D6 inhibitor = No CYP3A4 inhibitor = No Consensus Log *P*_o/w_ = 1.71
6	Pentadecanoic acid, 14-methyl-, methyl ester	C_16_H_32_O_2_	Antibacterial; Antifungal	Yes	Molecular weight = 256.42 g/mol GI absorption = High Water solubility = Moderately soluble Leadlikeness = No TPSA = 37.30 Å^2^ CYP1A2 inhibitor = Yes CYP2C19 inhibitor = No CYP2C9 inhibitor = No CYP2D6 inhibitor = No CYP3A4 inhibitor = No Consensus Log *P*_o/w_ = 5.07
7	n-Hexadecanoic acid	C_16_H_32_O	Antibacterial	Yes	Molecular weight = 240.42 g/mol GI absorption = High Water solubility = Moderately soluble Leadlikeness = No TPSA = 17.07 Å² CYP1A2 inhibitor = Yes CYP2C19 inhibitor = No CYP2C9 inhibitor = No CYP2D6 inhibitor = No CYP3A4 inhibitor = No Consensus Log *P*_o/w_ = 5.43
8	Octadecanoic acid, 9,10-dihydroxy, methyl ester	C_26_H_52_O_4_Si	Anti-inflammatory	Yes	Molecular weight = 456.77 g/mol GI absorption = Low Water solubility = Poorly soluble Leadlikeness = No TPSA = 55.76 Å^2^ CYP1A2 inhibitor = Yes CYP2C19 inhibitor = No CYP2C9 inhibitor = No CYP2D6 inhibitor = No CYP3A4 inhibitor = No Consensus Log *P*_o/w_ = 6.92
9	Phytol	C_19_H_38_O	Antibacterial	Yes	Molecular weight = 282.50 g/mol GI absorption = High Water solubility = Moderately soluble Leadlikeness = No TPSA = 20.23 Å^2^ CYP1A2 inhibitor = No CYP2C19 inhibitor = No CYP2C9 inhibitor = Yes CYP2D6 inhibitor = No CYP3A4 inhibitor = No Consensus Log *P*_o/w_ = 5.91
10	9,12,15-Octadecatrien-1-ol, (Z,Z,Z)-	C_18_H_32_	Antioxidant	Yes	Molecular weight = 248.45 g/mol GI absorption = Low Water solubility = Moderately soluble Leadlikeness = No TPSA = 0.00 Å^2^ CYP1A2 inhibitor = Yes CYP2C19 inhibitor = No CYP2C9 inhibitor = Yes CYP2D6 inhibitor = No CYP3A4 inhibitor = No Consensus Log *P*_o/w_ = 6.30
11	Octadecanoic acid	C_18_H_36_O	Antibacterial; Antifungal	Yes	Molecular weight = 268.48 g/mol GI absorption = Low Water solubility = Poorly soluble Leadlikeness = No TPSA = 17.07 Å^2^ CYP1A2 inhibitor = Yes CYP2C19 inhibitor = No CYP2C9 inhibitor = No CYP2D6 inhibitor = No CYP3A4 inhibitor = No Consensus Log *P*_o/w_ = 6.17
12	Bis(2-ethylhexyl) phthalate	C_23_H_36_O_4_	Antimicrobial; Cytotoxic	Yes	Molecular weight = 376.53 g/mol GI absorption = High Water solubility = Poorly soluble Leadlikeness = No TPSA = 52.60 Å^2^ CYP1A2 inhibitor = No CYP2C19 inhibitor = No CYP2C9 inhibitor = Yes CYP2D6 inhibitor = No CYP3A4 inhibitor = Yes Consensus Log *P*_o/w_ = 5.76
13	Methyl 9-cis,11-trans-octadecadienoate	C_18_H_32_O_2_	Anticarcinogenic; Anti-atherogenic	Yes	Molecular weight = 280.45 g/mol GI absorption = High Water solubility= Moderately soluble Leadlikeness = No TPSA =37.30 Å² CYP1A2 inhibitor = Yes CYP2C19 inhibitor = No CYP2C9 inhibitor = Yes CYP2D6 inhibitor = No CYP3A4 inhibitor = No Consensus Log *P*_o/w_ = 5.47

**Table 6:** Phytocompounds of *Acacia nilotica* showing ADMET properties for molecular docking.

Sr. No.	Phytocompounds	Lipinski Rule	ADMET Rule
1	Nonanoic acid	Yes	Molecular weight = 158.24 Consensus Log *P*_o/w_ =2.78 TP SA =17.07 Å² GI absorption = high Water solubility = soluble No; 1 violation: MW < 250 CYP1A2 inhibitor = No CYP2C19 inhibitor = No CYP2C9 inhibitor = No CYP2D6 inhibitor = No CYP3A4 inhibitor = No
2	Methyl 8-oxooctanoate	Yes	Consensus Log *P*_o/w_ = 2.70 TPSA = 26.30 Å² GI absorption = high Water solubility = soluble No; 2 violations: MW < 250, XLOGP3 > 3.5 CYP1A2 inhibitor = No CYP2C19 inhibitor = No CYP2C9 inhibitor = No CYP2D6 inhibitor = No CYP3A4 inhibitor = No
3	Nonanoic acid, 9-oxo-, methyl ester	Yes	Molecular weight = 172.26 Consensus Log *P*_o/w_ =3.13 TPSA =26.30 Å² GI absorption = high Water solubility = soluble No; 3 violations: MW < 250, Rotors > 7, XLOGP3 > 3.5 CYP1A2 inhibitor = No CYP2C19 inhibitor = No CYP2C9 inhibitor = No CYP2D6 inhibitor = No CYP3A4 inhibitor = No
4	Suberic acid monomethyl ester	Yes	Molecular weight = 172.22 Consensus Log *P*_o/w_ = 1.82 TPSA = 43.37 Å² GI absorption = high Water solubility = soluble No; 2 violations: MW < 250, Rotors > 7 CYP1A2 inhibitor = No CYP2C19 inhibitor = No CYP2C9 inhibitor = No CYP2D6 inhibitor = No CYP3A4 inhibitor =No
5	Nonanedioic acid, dimethyl ester	Yes	Molecular weight = 202.25 Consensus Log *P*_o/w_ =1.93 TPSA = 63.60Å² GI absorption = high Water solubility = soluble No; 2 violations: MW < 250, Rotors > 7 CYP1A2 inhibitor = No CYP2C19 inhibitor = No CYP2C9 inhibitor = No CYP2D6 inhibitor = No CYP3A4 inhibitor = No
6	2,2,4-Trimethyl-1,3-pentanediol diisobutyrate	Yes	Molecular weight = 272.38 Consensus Log *P*_o/w_ = 3.42 TPSA = 52.60 Å² GI absorption = high Water solubility = soluble No; 2 violations: Rotors > 7, XLOGP3 > 3.5 CYP1A2 inhibitor = No CYP2C19 inhibitor = Yes CYP2C9 inhibitor = No CYP2D6 inhibitor = No CYP3A4 inhibitor = No
7	1,2-Benzenedicarboxylic acid, bis(2-methylpropyl) ester	Yes	Molecular weight = 264.32 Consensus Log *P*_o/w_ = 3.30 No; 2 violations: Rotors > 7, XLOGP3 > 3.5 TPSA = 52.60 Å² GI absorption = high Water solubility= moderately soluble CYP1A2 inhibitor = Yes CYP2C19 inhibitor = Yes CYP2C9 inhibitor = No CYP2D6 inhibitor = No CYP3A4 inhibitor = No
8	Hexadecanoic acid, methyl ester	Yes	Molecular weight = 256.42 Consensus Log *P*_o/w_ = 5.20 TPSA = 37.30 Å² GI absorption = high Water solubility = moderately soluble No; 2 violations: Rotors > 7, XLOGP3 > 3.5 CYP1A2 inhibitor = Yes CYP2C19 inhibitor = No CYP2C9 inhibitor = Yes CYP2D6 inhibitor = No
9	n-Hexadecanoic acid	Yes	Molecular weight = 240.42 Consensus Log *P*_o/w_ = 5.43 TPSA = 17.07 Å² GI absorption = high Water solubility = moderately soluble No; 3 violations: MW < 250, Rotors > 7, XLOGP3 > 3.5 CYP1A2 inhibitor = Yes CYP2C19 inhibitor = No CYP2C9 inhibitor = No CYP2D6 inhibitor = No
10	Cyclic octa-atomic sulfur		Molecular weight = 256.52 Consensus Log *P*_o/w_ = TPSA =202.40 Å² GI absorption = Water solubility = soluble No; violation CYP1A2 inhibitor = CYP2C19 inhibitor = CYP2C9 inhibitor = CYP2D6 inhibitor = CYP3A4 inhibitor =
11	11-Octadecenoic acid, methyl ester	Yes	Molecular weight = 282.46 Consensus Log *P*_o/w_ =5.65 TPSA =37.30 Å² GI absorption = high Water solubility = moderately soluble No; 2 violations: Rotors > 7, XLOGP3 > 3.5 CYP1A2 inhibitor = Yes CYP2C19 inhibitor = No CYP2C9 inhibitor = Yes CYP2D6 inhibitor = No CYP3A4 inhibitor = No
12	9-Octadecenoic acid (z)-, methyl ester	Yes	Molecular weight = 284.48 Consensus Log *P*_o/w_ =5.93 TPSA = 37.30 Å² GI absorption = high Water solubility = poorly soluble No; 2 violations: Rotors > 7, XLOGP3 > 3.5 CYP1A2 inhibitor = Yes CYP2C19 inhibitor = No CYP2C9 inhibitor = No CYP2D6 inhibitor = No CYP3A4 inhibitor = No
13	Methyl stearate	Yes	Molecular weight = 284.48 Consensus Log *P*_o/w_ =5.93 TPSA =37.30 Å² GI absorption = high Water solubility = poorly soluble No; 2 violations: Rotors > 7, XLOGP3 > 3.5 CYP1A2 inhibitor = Yes CYP2C19 inhibitor = No CYP2C9 inhibitor = No CYP2D6 inhibitor = No
14	9-Octadecenoic acid, (E)-	Yes	Molecular weight = 266.46 Consensus Log *P*_o/w_ = 5.94 TPSA = 17.07 Å² GI absorption = low Water solubility = moderately soluble No; 2 violations: Rotors > 7, XLOGP3 > 3.5 CYP1A2 inhibitor = Yes CYP2C19 inhibitor = No CYP2C9 inhibitor = No CYP2D6 inhibitor = No CYP3A4 inhibitor = No
15	Methyl-9-cis,11-trans-octadecadienoate	Yes	Molecular weight = 393.52 Consensus Log *P*_o/w_ =3.72 TPSA = 86.23 Å² GI absorption = high Water solubility = soluble No; 3 violations: MW > 350, Rotors > 7, XLOGP3 > 3.5 CYP1A2 inhibitor = Yes CYP2C19 inhibitor = Yes CYP2C9 inhibitor = Yes CYP2D6 inhibitor = No CYP3A4 inhibitor = Yes
16	Octadecanoic acid	Yes	Molecular weight = 268.48 Consensus Log *P*_o/w_ = 6.17 TPSA = 17.07 Å² GI absorption = low Water solubility = poorly soluble No; 2 violations: Rotors > 7, XLOGP3 > 3.5 CYP1A2 inhibitor = Yes CYP2C19 inhibitor = No CYP2C9 inhibitor = No CYP2D6 inhibitor = No CYP3A4 inhibitor = No
17	Octadecanoic acid, 10-oxo-, methyl ester	Yes	Molecular weight = 298.46 TPSA =54.37 Å² GI absorption = high Water solubility = moderately soluble No; 2 violations: Rotors > 7, XLOGP3 > 3.5 CYP1A2 inhibitor = Yes CYP2C19 inhibitor = No CYP2C9 inhibitor = No CYP2D6 inhibitor = No CYP3A4 inhibitor =No
18	1H-pyrrol-1-yloxy,-3(aminocarbonyl)-2,5-dihydro-2,2,5,5-tetramethyl		Molecular weight= Consensus Log *P*_o/w_ = TPSA = Å² GI absorption = Water solubility = No; 1 violation: MW< CYP1A2 inhibitor = No CYP2C19 inhibitor = No CYP2C9 inhibitor = No CYP2D6 inhibitor = No CYP3A4 inhibitor = No

**Table 7:** Screened phytocompounds of *Ziziphus jujube*.

Sr. No.	Phytocompounds	Molecular Formula	Activity	ADMET Rule
1	2-Cyclopenten-1-one,3,4,4-trimethyl-	C_8_H_12_O	Antibacterial [18]	Molecular weight = 124.18 g/mol GI absorption = High Water solubility = Soluble Leadlikeness = No TPSA =17.07 Å² CYP1A2 inhibitor = No CYP2C19 inhibitor = No CYP2C9 inhibitor = No CYP2D6 inhibitor = No CYP3A4 inhibitor = No Consensus Log *P*_o/w_ = 1.80
2	Bis(2-ethylhexyl) phthalate	C_23_H_36_O_4_	Antimicrobial; Cytotoxic [19]	Molecular weight = 376.53 g/mol GI absorption = High Water solubility= Poorly soluble Leadlikeness =No TPSA =52.60 Å² CYP1A2 inhibitor = No CYP2C19 inhibitor = No CYP2C9 inhibitor = Yes CYP2D6 inhibitor = No CYP3A4 inhibitor = Yes Consensus Log *P*_o/w_ = 5.76

**Table 8:** Phytocompounds of *Acacia nilotica* screened on the basis of ADMET parameters for molecular docking.

Sr. No.	Phytocompounds	Activity	Lipinski Rule	ADMET Rule
1	Suberic acid monomethyl ester	Antibacterial [20]	Yes	Molecular weight = 172.22 Consensus Log *P*_o/w_ = 1.82 TPSA = 43.37 Å² GI absorption = high Water Solubility= soluble No; 2 violations: MW < 250, Rotors > 7 CYP1A2 inhibitor = No CYP2C19 inhibitor = No CYP2C9 inhibitor = No CYP2D6 inhibitor = No CYP3A4 inhibitor = No
2	2,2,4-trimethyl-1,3-pentanediol di-iso-butyrate	Antibacterial [2]	Yes	Molecular weight = 272.38 Consensus Log *P*_o/w_ = 3.42 TPSA = 52.60 Å² GI absorption = high Water Solubility = soluble No; 2 violations: Rotors > 7, XLOGP3 > 3.5 CYP1A2 inhibitor = No No CYP2C19 inhibitor = Yes No CYP2C9 inhibitor = No No CYP2D6 inhibitor = No No CYP3A4 inhibitor = No No

**Table 9:** Protein screening based on mode of action for molecular docking of *Ziziphus jujube*.

Sr. No.	Proteins	Mode of Action	Reference
1	5L3J DNA Gyrase *E. coli* protein	DNA Gyrase is proven to be validated targets in the design of novel antibacterial drugs	[21]
2	TSST-1 2QIL *S. aureus* protein	TSST-1 is a super antigen that stimulates release of cytokines, producing leakage of endothelial cells at low concentrations and a cytotoxic effect to the cells at high concentrations	[22]

**Table 10:** Mode of action of *S. aureus* and *E. coli* proteins for molecular docking of *Acacia nilotica*.

Sr. No.	Proteins	Mode of action	Reference
1	1BXW Outer membrane protein A *E. coli* protein	Outer membrane protein enables intracellular survival, evasion from the body’s defense.	[23]
2	3FV5 Topoisomerase IV *E. coli* protein	Topoisomerase IV helps in creating pores in membranes of host cells (cell lysis) and catalyzing a DNA double-strand break.	[24]
3	2QIL Toxic shock syndrome toxin-1 *S. aureus* protein	TSST-1 is a super antigen that stimulates release of cytokines, producing leakage of endothelial cells at low concentrations and a cytotoxic effect to the cells at high concentrations.	[22]
4	3SEB Enterotoxin B *S. aureus* protein	Enterotoxin B helps in the stimulation of cytokine release and inflammation.	[25]

**Table 11:** Binding energies of ligands of plant extracts with bacterial proteins of *E. coli* and *S. aureus*.

*Ziziphus jujube*					
Sr. No.	Ligands	*E. coli* Protein with PDB Id 5L3J		*S. aureus* Protein with PDB Id 2QIL	
1.	2-Cyclopenten-1-one,3,4,4-trimethyl-	−4.8 kcal/mol		−5.3 kcal/mol	
2.	Bis(2-ethylhexyl) phthalate	−5.9 kcal/mol		−7.1 kcal/mol	
**Acacia nilotica**					
**Sr. No.**	**Ligands**	***E. coli* proteins**		***S. aureus* proteins**	
		**1BXW**	**3FV5**	**2QIL**	**3SEB**
1.	Suberic acid monomethyl ester	−5.9 kcal/mol	−5 kcal/mol	−5.1 kcal/mol	−5.1 kcal/mol
2.	2,2,4-trimethyl-1,3-pentanediol di-iso-butyrate	−6.5 kcal/mol	−5.3 kcal/mol	−5.2 kcal/mol	−5.2 kcal/mol

## Data Availability

All the data is available in article.

## Supplementary Materials

The following supporting information can be downloaded at: https://www.mdpi.com/article/10.3390/microorganisms11102450/s1, Figure S1. Molecular identification (amplicons of PCR on agarose gel) of *S. aureus* by targeting *nuc gene*. M indicates a marker of 1 kb, 1–5 are samples, P means positive control. Figure S2 Molecular identification of *E. coli* targeting 23S RNA gene. M indicates marker of 1000 bp, 1–3 are samples, P indicates positive control, N indicate negative control
