# Supplementary material for: Validating Interactions of Pathogenic Proteins of Staphylococcus aureus and E. coli with Phytochemicals of Ziziphus jujube and Acacia nilotica
Source: Microorganisms. 2023 Sep 29;11(10):2450. doi: 10.3390/microorganisms11102450 (PMC10609126; doi:10.3390/microorganisms11102450)
Supplement: Supplementary file 1 [file microorganisms-11-02450-s001.zip › microorganisms-2549881-supplementary.pdf]

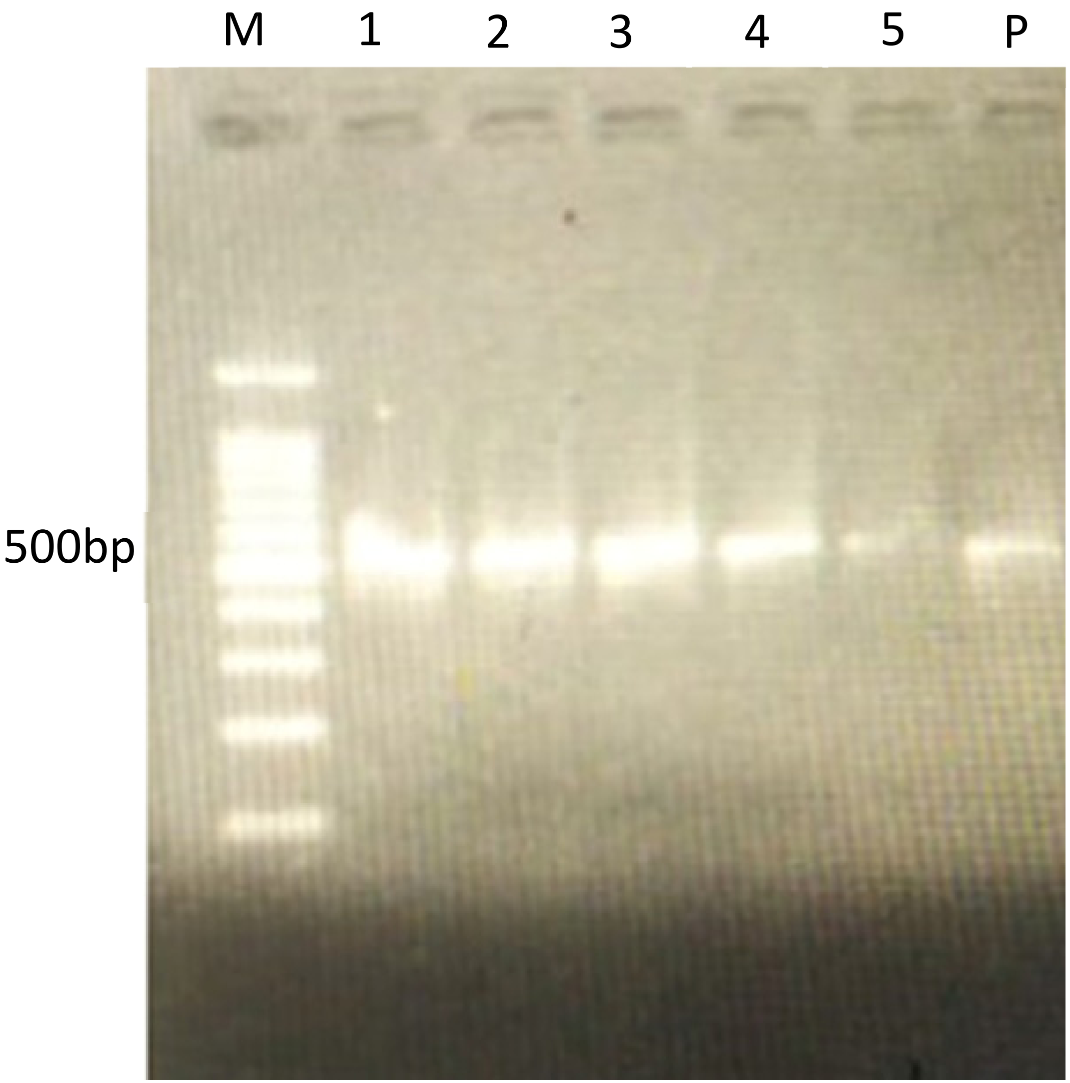

Figure S1. Molecular identification (amplicons of PCR on agarose gel) of *S. aureus* by targeting *nuc gene*

M indicates a marker of 1kb, 1-5 are samples, P means positive control

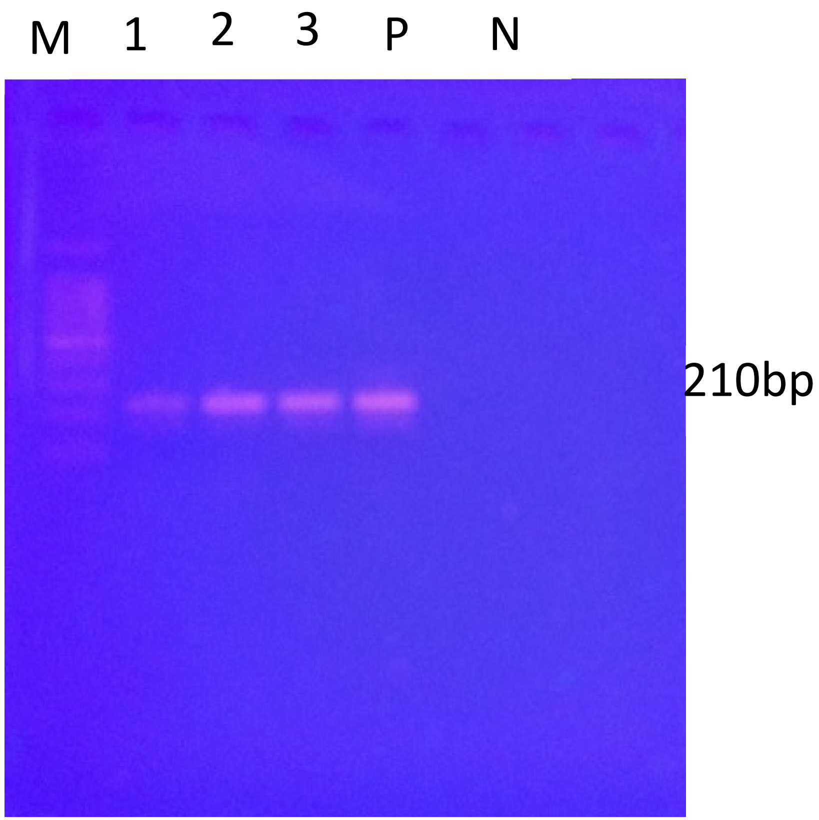

Figure S2 Molecular identification of *E. coli* targeting 23S RNA gene
M indicates marker of 1000 bp, 1-3 are samples, P indicates positive control, N indicate negative control
